# Application of digital health technologies to substance use reduction among students in higher education institutions: A scoping review

**DOI:** 10.12688/f1000research.163565.1

**Published:** 2025-04-24

**Authors:** Mistire Teshome Guta, Fira Abamecha, Demuma Amdisa, Kalkidan Hassen Abate Abate

**Affiliations:** 1 Department of Nursing, Wolaita Sodo University, Sodo, Southern Nations, Nationalities, and People's Region, Ethiopia; 2 College of Public Health and Medical Sciences, Jimma University, Jimma, Oromia, Ethiopia

**Keywords:** Digital health technology, substance use reduction, students in higher education institutions, scoping review.

## Abstract

Substance use among college and university students is associated with significant health issues, academic struggles, and premature death. This scoping review explores the potential of digital health interventions, including internet-based and mobile platforms, to reduce substance use. A comprehensive search across databases such as PubMed, PsycINFO, Scopus, and Google Scholar identified 11 eligible studies conducted across seven countries between 2013 and 2025. These studies focused primarily on alcohol use and included digital health tools like instant messaging, Telegram applications, text messaging, and web-based interventions. The results suggest that digital health technologies can effectively motivate college students in low- and middle-income countries (LMICs) to reduce or abstain from psychoactive substance use. However, there is a notable research gap in evaluating the effectiveness and feasibility of these tools, especially mobile text messaging, which remains one of the most widely used methods in LMICs. The review highlights the need for further research, including systematic reviews and meta-analyses, to better understand the impact of digital health interventions on substance use reduction and to develop evidence-based programs for behavior change.

## Highlight

Substance use is an escalating issue among young people, particularly in LMICs, where rapid economic, social, and cultural shifts are fostering an environment conducive to increased substance use. College life introduces unique challenges such as separation from family, the formation of new social networks, and academic pressures that can further contribute to substance use. There is a significant gap in research regarding digital health interventions aimed at reducing substance use among college students in low- and middle-income countries. To address this gap, we conducted a scoping review of existing literature, summarizing studies that have evaluated digital health technologies for substance use reduction within this population. Our review focuses on identifying the types of digital health technologies utilized their intended purposes, and their reported outcomes.

## Introduction

Young adulthood is a crucial stage of life characterized by significant emotional, educational, vocational, and social transitions. This period marks the adoption of adult roles, responsibilities, and the development of social skills. For many college and university students, this phase is particularly transformative, involving a shift towards greater independence from parental supervision, exposure to new social and academic pressures, and an environment where the use of intoxicating substances particularly alcohol is widespread. ^1, 2^ Psychoactive substances, which include compounds that alter mental processes such as perception, consciousness, cognition, mood, and emotions, encompass a wide range of substances like alcohol, marijuana, nicotine, and khat. While the term “psychoactive” doesn't always indicate dependence, it is frequently used to describe substances that can lead to addiction or misuse, with terms like “substance use” or “substance abuse” often associated with it. ^3^ Unfortunately, substance use among young adults has contributed to an increase in psychosocial issues, ^4^ and individuals with substance use disorders are statistically more likely to experience mental health disorders and premature mortality compared to those without such conditions. ^5, 6^

For many young people, college enrollment is a pivotal transition from adolescence to adulthood, often associated with an increased likelihood of substance use. While attending college was once thought to be a protective factor against substance abuse, recent trends have shown a concerning rise in substance use disorders among university students. ^7^ These students are considered a high-risk group, ^8^ with both alcohol consumption and the use of legal and illegal drugs increasing globally, ^8, 9^ particularly in low- and middle-income countries (LMICs). In countries like Ethiopia, 46.74% individuals aged 18-24 engage in substance use, with alcohol (36.34%) being the most commonly consumed psychoactive substance. ^10^ The move from high school to college also brings heightened peer interactions and exposure to social norms that can influence behavior. ^9^ Peers often directly encourage substance use or subtly shape perceptions of acceptable behavior, increasing the likelihood of risky behaviors such as smoking, drinking, and using drugs. ^11^

The global impact of substance use is vast, contributing significantly to the disease burden worldwide. Alcohol and illicit drugs account for a large portion (5.4%) of the global burden of disease, with cigarette use alone (3.7%) responsible for a substantial percentage. ^12^ Despite the increasing number of individuals affected by substance use disorders estimated at 64 million people globally only a small fraction (only 1 in 11 people) receives the necessary treatment. ^13^ Young people are particularly vulnerable to the negative effects of substance use, which can disrupt their health, academic performance, social relationships, and future career prospects. ^14– 16^ University students face unique challenges, as the combination of newfound independence, the pressures of academic life, and exposure to a range of social influences can heighten the risk of engaging in substance use. ^6, 15, 17– 20^ The challenges are compounded by the changing cultural norms and rising rates of substance misuse in developing countries, including parts of LMIC, ^16– 18, 21^ where alcohol, tobacco, and other substances are increasingly common in schools and universities. ^22^ As a result, the risk of substance abuse is enhanced in academic settings. ^14^

In many LMIC nations, the increasing prevalence of substance use is linked to significant social and economic transformations. ^20^ Psychoactive substances such as alcohol, cannabis, tobacco, and even harder drugs like heroin and cocaine are often found in educational institutions. ^23^ Alcohol and cigarettes are especially problematic, as they are commonly seen as “gateway drugs” that lead to the use of more harmful substances. ^6, 14, 16^ Regular substance use is associated with several negative outcomes for students, including poor academic performance, increased absenteeism, and higher dropout rates. ^24– 27^ Moreover, substance use can lead to long-term health and psychiatric issues, significantly impacting students’ futures. ^28, 29^

Digital health interventions represent a promising avenue for addressing these issues among university students. Digital health, which involves the use of information and communication technologies to promote physical, mental, and social well-being, includes a wide range of tools such as websites, mobile applications, and other technology-based solutions aimed at disease prevention and health promotion. ^30^ Universities worldwide are increasingly embracing these digital solutions to improve student health. Examples of digital health initiatives include mobile apps for reducing substance use, ^31^ promoting healthy behaviors like physical activity, ^32, 33^ and improving mental health. ^34– 36^ While much of the research on these digital health tools has been conducted in experimental settings, particularly in Western countries, the landscape is evolving, with many new digital health resources becoming available. ^37^

However, there is a significant gap in research regarding the use of digital health interventions to reduce substance use among university students in LMICs. The existing literature is limited in terms of evaluating the effectiveness of these digital tools in such settings. A scoping review of the available literature on this topic aims to fill this gap by identifying the types of digital health technologies used for substance use reduction, the purposes of these tools, and the outcomes they produce. Unlike systematic reviews, which provide critically appraised and synthesized findings, a scoping review takes a broader approach, exploring the range of digital health technologies currently in use. This review is essential for understanding how digital health interventions can be optimized to address the growing issue of substance use among university students in LMICs, helping to guide future research and intervention strategies in this area.

## Methods

We used the procedures recommended by the technique for scoping reviews ^38^ and followed the Joanna Briggs Institute Preferred Reporting Items for Systematic Review and Meta-Analyses Extension for Scoping Reviews (PRISMA-ScR) checklist and reporting guideline ^39^ (S1, Supporting Information). The preliminary protocol was with the PI. In summary, we took the following steps: (1) identifying the research question; (2) identifying relevant studies; (3) selecting studies; (4) charting the data; and (5) collecting, summarizing, and reporting the results.

The process for developing a research question on digital health technologies for substance use reduction among higher education students in low- and middle-income countries (LMICs) was outlined in several stages. In **Stage 1**, the research question was designed to explore various digital health technologies, their specific elements, and their effectiveness in reducing substance use. The key questions guiding the study included identifying the technologies used, understanding their key features, and assessing their reported outcomes in the target population.

**Stage 2** focused on identifying relevant studies, with a search strategy that included a review of original studies published up to March 2025. The search prioritized literature on digital health technologies for substance use prevention, particularly those influencing policy and practice. Major global databases such as Scopus, PubMed, and PsycINFO were systematically searched using Boolean operators, incorporating keywords related to substance use and digital health technologies. A comprehensive search strategy was used, with a combination of database searches, manual reviews on platforms like Google Scholar, and backward and forward citation searches. The eligibility of studies was based on the Population, Concept, Context (PCC) framework, which ensured that studies focused on students from LMICs, used digital health technologies, and addressed substance use exposure. We used the World Bank Country and Lending Groups list of LMICs for 2019–2020. ^40^ Inclusion criteria were clearly defined, while studies like protocols, editorials, and systematic reviews were excluded.

**Stage 3** involved selecting studies through a two-stage screening process. Initially, duplicates were removed, and titles were screened to identify relevant studies for abstract review. In the second stage, abstracts were independently reviewed for inclusion in the full-text analysis. Any disagreements in article selection were resolved through a consensus decision involving a third investigator.

In **Stage 4**, the data extraction process began with the preparation of a comprehensive form to capture information from the included articles, such as study design, sample size, types of substances used, and the digital health technologies employed. Two authors conducted the data extraction, with third-party involvement if discrepancies occurred. This data was organized into a spreadsheet and will be included as a supplemental file.

Finally, in **Stage 5**, the results were collated, summarized, and reported. The findings were categorized into emergent themes such as publication trends, research design, substance types, technology used, outcomes assessed, and gaps in the research. The scope of digital health technologies for substance use reduction among LMIC university students was mapped, providing insight into existing methodologies and areas for future investigation.

### Assessment of study quality

To assess the methodological quality of the included studies, we used the Joanna Briggs Institute (JBI) Critical Appraisal Tools. This tool was selected because it is well-suited for evaluating the diverse range of study designs included in scoping reviews, including quantitative, qualitative, and mixed methods research.

The quality of the studies was assessed across several domains, including methodological rigor, validity, relevance to the research question, and transparency of reporting. The results of the quality appraisal were summarized descriptively to provide an overview of the methodological strengths and weaknesses of the included studies. However, no studies were excluded based on quality to maintain the inclusive nature of the scoping review process.

## Results

### Search results using PRISMA-ScR

**Figure 1.**
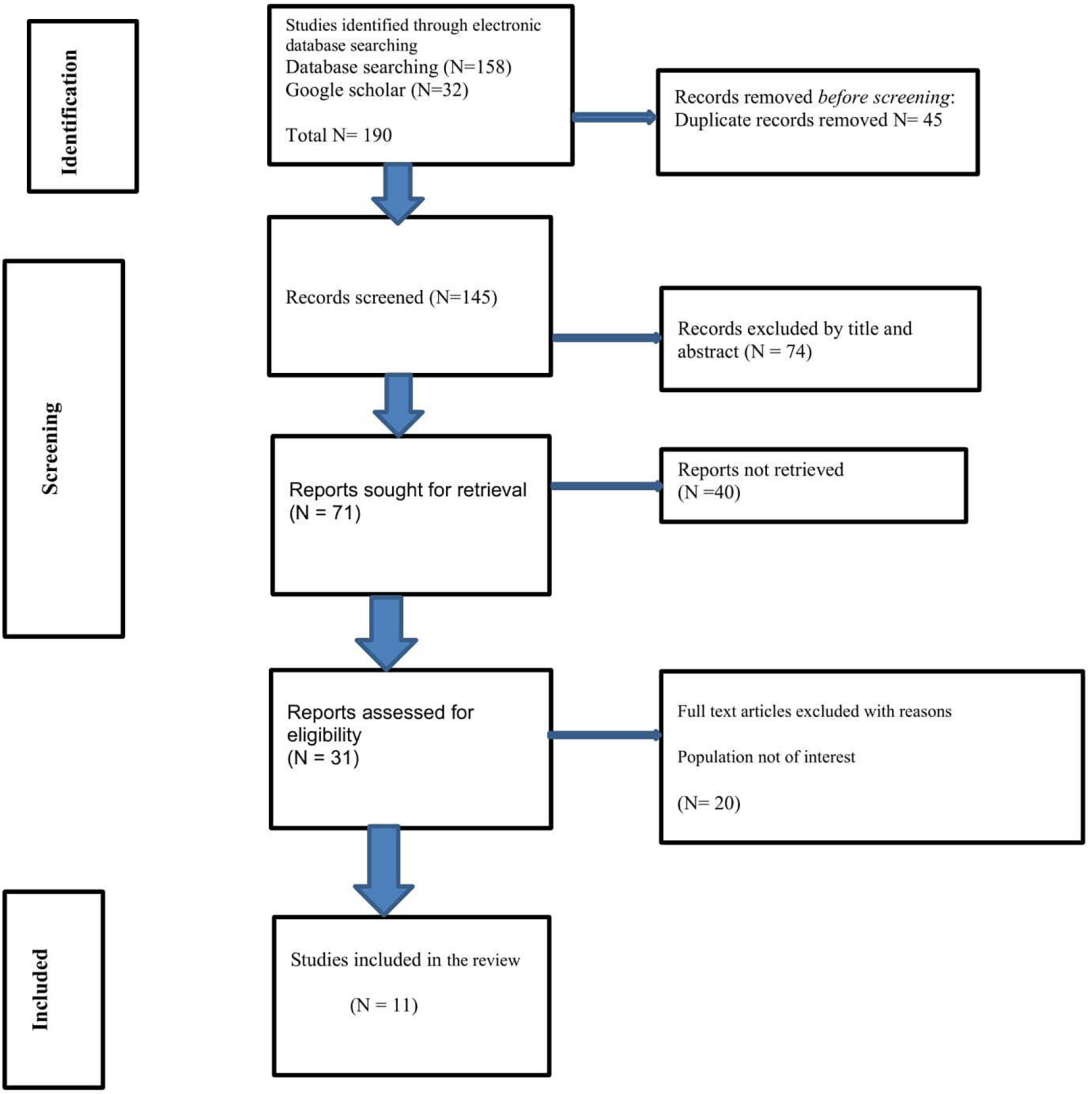
PRISMA Flowchart describing the selection of studies mapping existing literature on digital health technology for substance use reduction in young adult college or university students in the LMIC.

Our search encompassed four electronic databases: PubMed, ^41^ PsycINFO via APA, ^42^ Scopus, ^43^ and Google Scholar, ^32^ which resulted in an initial pool of 190 articles. After removing 45 duplicates, we screened the remaining 145 articles based on their titles and abstracts. This process led to the exclusion of 74 publications that did not meet our inclusion criteria. Additionally, we included one study from the reference lists of reviewed articles ^43^ and another from Google scholar. ^44^ Following a full-text review of the remaining 31 articles, 11 studies were selected for data extraction and analysis. The review process ensured that only those studies meeting the rigorous inclusion criteria were included in the final analysis ( Figure 1). This method of review was conducted on the basis of a predetermined protocol in accordance with the Preferred Reporting Items for Systematic reviews and Meta-Analyses (PRISMA) standards for scoping reviews ^39^ (S1- PRISMA Checklist). Lastly, we searched for grey literatures, but none met the inclusion criteria.

### Characteristics of the studies

The review identified 11 original, peer-reviewed studies published between 2013 and 2025 that focus on the use of digital health technology for substance use reduction in low- and middle-income countries (LMICs), meeting the inclusion criteria. Among these, five articles (45.5%) were published in the last five years (2020-2025), with 2019 having the highest number of relevant publications (n = 3, 27.3%). The years 2018 and 2025 each had two relevant publications (n = 2, 18%), while 2015 and 2020 had one publication each. Despite the growing interest in this field, the overall number of studies remains insufficient, indicating a lack of original, peer-reviewed research on this topic within LMIC contexts. The earliest study meeting the eligibility criteria was conducted in 2015. The review includes studies from various years, including three studies from 2019, two from 2018, 2023, and 2024, and one each from 2020 and 2015.

Of the 11 included primary studies, seven employed quantitative methodologies using randomized controlled trial (RCT) designs, ^44– 50^ while one study utilized a quasi-experimental approach, ^51^ one used an interventional study with a single group, ^43^ another employed a cross-sectional methodology, ^52^ and one article adopted a qualitative approach. ^53^ The studies spanned five countries: Brazil had the highest representation, ^45– 48^ contributing four studies, followed by Hong Kong with two, ^49, 53^ and Iran, ^51^ Korea, ^50^ and Vietnam, ^52^ each contributing one study. The participants in the studies were college or university students aged 18 years or older. For instance, in Brazil, ^45, 46^ the studies focused on college drinkers aged 18–30 who reported alcohol use in the past three months. In Hong Kong, ^47^ one study involved participants aged 18 or older who had consumed alcohol in the last 12 months, while another study focused on students at risk for alcohol use disorder. ^49^ In Tehran, ^51^ a study included 130 students living in dormitories to assess smoking prevention behaviors. Additional studies included participants from Turkey, India, Iran and other countries ( Figure 3) (Tables 1 and 2). ^74^

The studies employed a range of research designs to evaluate interventions targeting substance use, showcasing diverse methodological approaches. One study ^44^ conducted a mixed-methods cluster randomized controlled trial, integrating both quantitative and qualitative elements. Several studies used pragmatic RCTs, ^47^ including a dismantling design ^46^ and standard approaches. ^45^ Other studies utilized quasi-experimental designs, ^43^ cross-sectional surveys, and semi-structured interviews to assess intervention effectiveness. The sample sizes in these studies varied widely, from a small group of 20 university students to larger cohorts, such as one study analyzing data from 5,476 participants. Other studies included 931, 4,460, and 772 participants, with one focusing on 458 participants, and another examining a cohort of 191 individuals. The studies also demonstrated a mix of large-scale and smaller, targeted research efforts, illustrating the diverse strategies employed to explore substance use in different contexts (Table 1). ^74^

**Figure 2.**
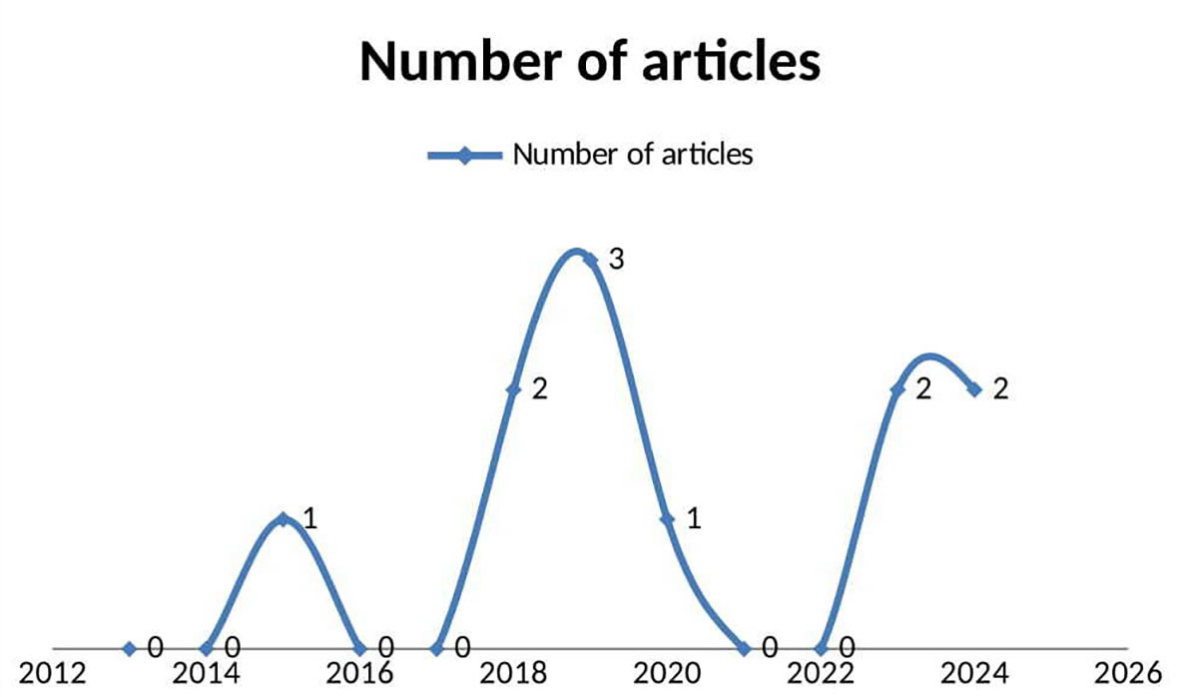
Lome graph showing articles published per year (publication trends as of time of data collection).

The earliest study meeting our eligibility criteria was conducted in 2015. ^48^ In the review: three studies were identified in 2019, ^43, 45, 50^ two primary studies each were identified of the following years: 2018, ^51, 52^ 2023 ^44, 53^ and 2024. ^47, 49^ One study was found in, 2020 ^46^ and 2015 ^48^ ( Figure 2).

**Figure 3.**
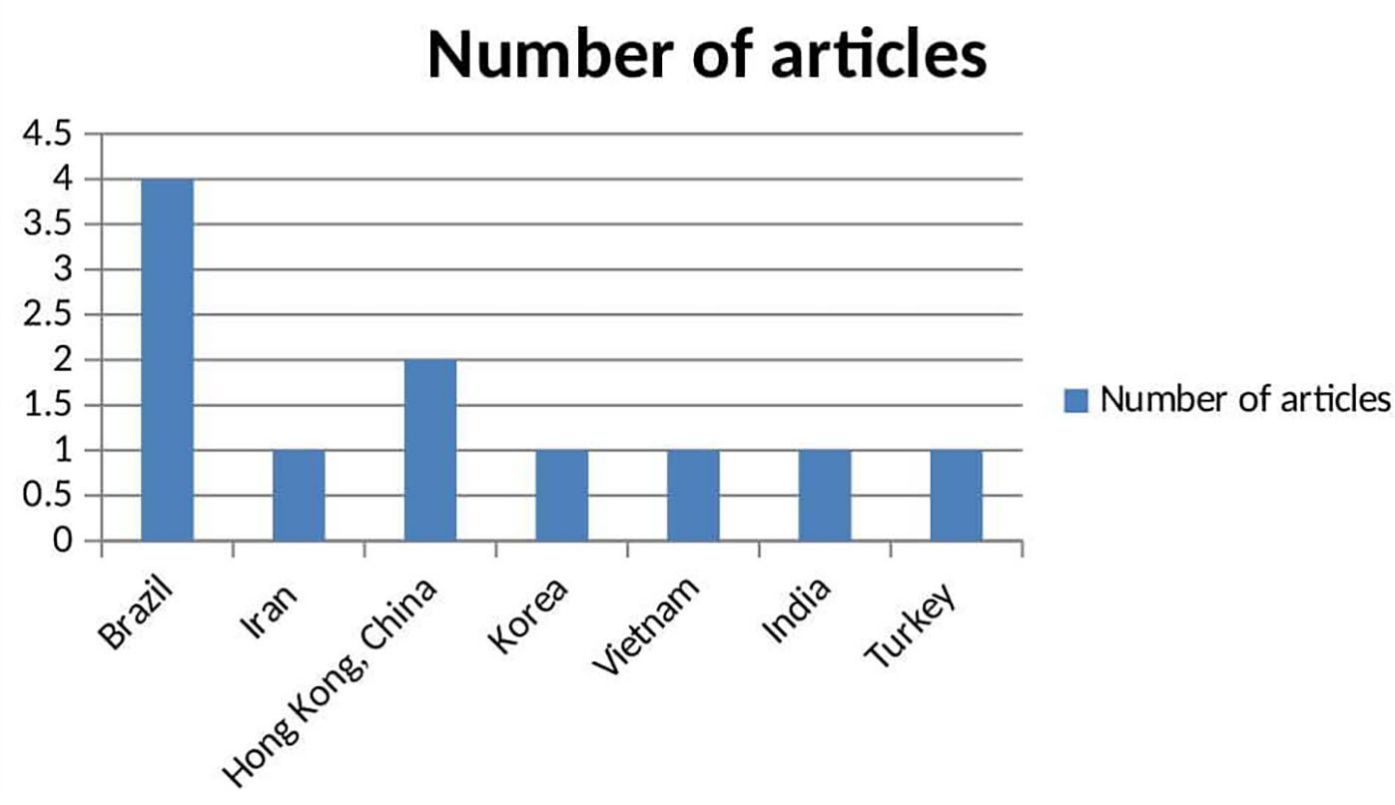
Bar chart showing the distribution of articles per countries.

The studies in the review involved a wide range of sample sizes, further highlighting the diversity of research approaches. One study ^46^ analyzed data from a large cohort of 5,476 participants, while another ^47^ included 931 individuals, and a third study ^45^ involved 4,460 participants. One study ^49^ had 772 participants, whereas another ^53^ focused on a smaller group of 20 university students. Additionally, one study ^48^ involved 458 participants, while another ^44^ examined a sample of 191 individuals (Table 2). ^74^

One study ^43^ initially recruited 433 participants, completing their study with 314 individuals. Another study ^51^ focused on a smaller group of 130 participants, while a separate study ^50^ included a larger cohort of 1,496 individuals. Finally, one study ^52^ worked with a moderately sized cohort of 1,082 participants. Together, these studies reflect a balanced mix of large-scale and smaller, targeted research efforts, illustrating the multifaceted strategies employed to explore and address substance use across diverse populations and contexts (Table 2). ^74^

### Type of digital health technologies

The most commonly used digital health technology in the included studies was the web-based personalized normative feedback (PNF) intervention, implemented in three studies. ^45– 47^ One study ^50^ utilized a web-based intervention called the Brief Empowerment Program for Alcohol-use Monitor (BEAM), which integrated PNF and motivational interviewing (MI) components. Other technologies included web portals or Android applications, ^44^ a web-based program, ^43^ e-health interventions, ^52^ a computer-based intervention, ^48^ the Telegram application, ^51^ instant messaging apps, ^53^ and mobile chat-based instant messaging ^49^ (Table 1). ^74^

### Substance use reduction

The studies in the review employ a range of methods to define and assess substance use, focusing on alcohol and tobacco, and use validated tools like AUDIT and ASSIST to measure consumption patterns and associated risks. The common thread is the evaluation of substance use behaviors to identify risky patterns and inform potential interventions.

The majority of included primary studies focused on alcohol use, with seven articles ^44– 47, 49, 50, 53^ examining this substance. Three articles investigated tobacco smoking ^43, 51, 52^ and one explored broader substance use, encompassing tobacco, alcohol, marijuana, cocaine, amphetamine-stimulants, inhalants, sedatives, hallucinogens, opioids, and other substances. ^48^ The definition of substance use across the studies is largely consistent, revolving around alcohol consumption or smoking behavior, though the timeframes and criteria vary.

For alcohol use, several studies define it based on consumption in the previous months or year, ^46, 47^ who focus on alcohol use in the past 3 months and 12 months, respectively, using the AUDIT-C tool to assess the frequency and quantity of consumption. Other studies in the review, ^45, 49^ expand the definition to include hazardous drinking patterns and risk levels, which are measured using the AUDIT tool. Some studies, ^50^ set specific cutoffs for identifying problematic alcohol use (e.g., AUDIT-C scores of 4 for men and 3 for women).

Tobacco use is similarly defined as the act of smoking or using tobacco products. For instance studies like, ^43, 51^ focus on smoking behaviors, with defining smoking as using at least one cigarette a day and the Health Belief Model to assess smoking behaviors and perceptions of risk. Additionally, studies like ^48^ examine both alcohol and drug use, including tobacco, using the ASSIST screening tool.

Measurement tools and criteria are generally based on well-established scales, including AUDIT, AUDIT-C, and ASSIST, which assess the severity of substance use and associated risks. For instance, the AUDIT-C tool is commonly used to evaluate alcohol consumption, while the ASSIST tool is employed to assess alcohol, tobacco, and other drug use. These tools help to identify hazardous drinking behaviors, smoking habits, and potential substance use disorders, often through self-reported surveys (Table 1). ^74^

### Purpose and digital health strategies

The included studies explored various purposes related to the digital health technology: Two studies ^45, 46^ aimed to reduce alcohol use and alcohol-related consequences. Three studies focused on reducing alcohol use specifically, ^47, 49, 53^ while one study ^50^ aimed to prevent problematic alcohol use and the other one on digital screening and brief intervention for alcohol misuse. ^44^ Two articles addressed tobacco smoking cessation, ^43, 52^ and another focused on smoking preventive behaviors. ^51^ Finally, one study aimed to reduce overall substance use involvement ^48^ (Table 1). ^74^

The studies in the review explored various digital interventions aimed at address substance use, particularly alcohol and smoking, in college and university settings. One study ^44^ developed a digital screening and brief intervention for alcohol use, delivered through an interactive digital platform tailored to help college students reduce their alcohol consumption. Another study ^43^ designed a web-based smoking cessation program that offered educational tools, resources, personalized counseling, and progress tracking to support quitting (Table 1). ^74^

One study created an online platform providing students with customized feedback on their alcohol consumption compared to peer norms, delivered through self-paced web sessions. ^46^ In a similar vein, another study ^45^ implemented a web-based intervention for Brazilian college students focusing on motivational strategies to reduce alcohol use. A different study ^47^ developed a personalized normative feedback system to address alcohol-related misperceptions among students using an online platform. Additionally, one study ^52^ introduced an e-health intervention to promote smoking cessation through educational content, personalized counseling, and behavior change strategies delivered via the internet and mobile platforms (Table 1). ^74^

Another study ^48^ evaluated a computer-based intervention with three groups: one receiving computerized screening and motivational intervention, another receiving non-computerized screening with motivational intervention and a control group undergoing assessment only. One study ^51^ applied an educational intervention based on the Health Belief Model and health literacy principles, incorporating lectures, discussions, and health education materials (Table 1). ^74^

One study ^53^ used instant messaging apps to deliver alcohol reduction interventions, emphasizing personalized communication and engagement. In another study ^49^ employed the brief interventions using either instant messaging (IM) or text messaging (SMS) to support alcohol reduction efforts. Lastly, a study ^50^ implemented a web-based screening and brief intervention targeting problematic alcohol use, providing tailored feedback through an online platform (Table 1). ^74^

These interventions highlight a wide range of strategies, including personalized feedback, counseling, motivational techniques, and behavioral support, delivered through digital platforms such as web-based systems, mobile apps, and messaging services to address substance misuse among college students (Table 1). ^74^

### Dose and duration of the Interventions

The studies in the review implemented various interventions with differing doses and durations. One study ^44^ conducted a single digital session that combined screening, feedback, and motivational interviewing, followed up over a period of 3 months. Another study ^43^ provided participants access to a gradual, content-based program with weekly follow-ups or reminders over 6 months. A third study ^46^ implemented a single-session, web-based Personalized Normative Feedback (PNF) intervention for alcohol use with a follow-up duration of 6 months, while another study ^45^ also used a single web-based session featuring personalized feedback over the same duration. Additionally, one study ^47^ enhanced its web-based PNF intervention with booster sessions, maintaining a 6-month timeframe (Table 1). ^74^

A separate study ^52^ focused on smoking habits, offering personalized feedback and encouraging participants to set quit dates and track their progress. Another study ^48^ implemented a single-session tailored intervention addressing substance involvement with feedback components, with a follow-up over 3 months. One study ^51^ implemented an electronic educational intervention via six sessions delivered through the Telegram application over 3 months, while the control group received no intervention (Table 1). ^74^

Another study ^53^ explored participants’ perceptions of intervention doses, concentrating on qualitative insights into app usage without implementing a specific dose. Meanwhile, a separate study ^49^ featured two intervention groups: one receiving chat-based instant messaging support for alcohol reduction, and the other receiving SMS messages on general health topics, both over 3 months. Lastly, one study ^50^ delivered a single-session intervention with personalized feedback on alcohol use, with a follow-up period of 4 weeks (Table 1). ^74^

### Outcomes of digital categories

The review of the included primary studies revealed diverse outcomes for the digital intervention categories. One study ^46^ found no evidence supporting the intervention's effectiveness, while another ^45^ demonstrated its success in reducing alcohol use. A third study ^47^ observed that the intervention was effective in reducing alcohol consumption for one month but showed no lasting impact thereafter. Another study ^52^ highlighted the feasibility of integrating e-health interventions with traditional clinical or telephone-based models. One study ^48^: reported effectiveness in reducing alcohol use, low effectiveness for marijuana, and inconsistent results for tobacco and other drugs. An educational intervention using the Telegram application, grounded in the Health Belief Model (HBM) and Health Literacy (HL), was effective in promoting smoking prevention behaviors among university students. ^51^ Instant messaging interventions were found to be highly acceptable. ^53^ Two studies ^49, 50^ demonstrated the effectiveness of the mobile chat-based instant messaging and web-based interventions specifically on-BEAM, which incorporates personalized normative feedback (PNF) and motivational interviewing (MI) components. Additionally, one study ^44^ found that a digital screening and brief intervention for alcohol misuse was acceptable, feasible, and potentially effective among college students from low-resource settings. Lastly, another program ^43^ successfully helped students quit smoking, enhanced their self-efficacy, and facilitated the process of change toward smoking cessation (Table 1). ^74^

### Outcomes measured

The studies included in the review assessed a range of outcomes related to substance use interventions, particularly focusing on alcohol and smoking behaviors. Several studies measured changes in alcohol consumption and related behaviors. For instance, one study ^46^ evaluated self-reported changes in alcohol use and associated behaviors, while a later study ^47^ specifically measured reductions in weekly alcohol consumption and drinking-related consequences. Similarly, one study ^45^ assessed alcohol use through AUDIT scores, drink counts, and consequences. One study in Hong Kong ^49^ also focused on alcohol use reduction, while another study ^53^ examined the feasibility, acceptability, and perceptions of using instant messaging apps for reducing alcohol consumption (Table 2). ^74^

One study ^48^ evaluated substance involvement reduction for alcohol, tobacco, and cannabis, reflecting a broader scope of intervention. Another study ^44^ examined changes in alcohol use behaviors and associated risks, providing insights into behavioral outcomes (Table 2). ^74^

Smoking-related outcomes were also a focus of several studies. One study ^43^ measured smoking cessation rates, self-efficacy, and the process of change, while another study ^51^ evaluated the adoption of smoking prevention behaviors using the Health Belief Model combined with health literacy principles. One study ^52^ assessed smoking prevalence, quit attempts, and the willingness to pay for cessation apps (Table 2). ^74^

Finally, one study ^50^ investigated reductions in AUDIT-C scores as a measure of alcohol use. Collectively, these studies highlight a variety of outcome measures, including behavioral changes, feasibility and acceptability of interventions, and the effectiveness of specific strategies to reduce substance use (Table 2). ^74^

In general the review targeting alcohol and smoking behaviors among university students, predominantly aged 15 and above, revealed diverse methodologies and outcomes based on context and population. Three studies in the review evaluated Personalized Normative Feedback (PNF) interventions in Brazil, finding them effective in reducing alcohol consumption, especially in motivated participants, though effectiveness diminished with low motivation or longer follow-ups. ^45– 47^ Similarly, one study demonstrated that instant messaging interventions reduced short-term alcohol use in Hong Kong, ^49^ while their qualitative study highlighted user acceptance and preference for personalized, private digital interventions. ^53^ One study in the review reported on computerized screening and motivational interventions that effectively reduced substance involvement, particularly alcohol, though challenges remained in cannabis use. ^48^ The other study in India utilized digital tools, showing feasibility and potential effectiveness despite limited generalizability. ^44^ Smoking cessation by two studies in Turkey and Iran ^43, 51^: leveraged web-based and educational interventions integrating behavioral models, improving cessation rates and preventive behaviors. Lastly, one study in the review explored e-health interventions in Vietnam, noting their feasibility but emphasizing the need for reliable information to enhance uptake ^52^ (Table 2). ^74^

## Discussion

This scoping review aimed to explore various digital health technologies to reduce substance use, focusing on their applications, specific elements, and effectiveness among college and university students in low- and middle-income countries (LMICs). The findings reveal a significant lack of peer-reviewed articles on this topic, particularly within the last five years (2020–2025). Of the few studies identified, only four focused on LMICs, with none from Africa, highlighting a critical research gap. The earliest study included was from 2015, ^48^ emphasizing the need for more primary research, despite the increasing availability of information technology in recent years. ^54, 55^ This review collectively highlights the promise of tailored digital and educational interventions, albeit with varying levels of success influenced by participant motivation, cultural contexts, and methodological limitations.

Brazil had the most studies included in the review, likely due to its significant internet growth and widespread smartphone penetration, especially among individuals aged 18–55. ^56^ However, other LMICs face challenges like limited internet access, illiteracy, worsening poverty, and a lack of research from Africa. ^57, 58^ Notably, no studies from Nigeria—a country with the highest number of internet users in Africa were included, suggesting potential issues with resource allocation and prioritization of research on substance use disorder (SUD) interventions, ^42^ lacked representation. Despite these challenges, our findings suggest that digital health interventions have the potential to decrease substance use in LMICs. However, the effectiveness of these interventions may not be widely reported or published, highlighting the need for more research and dissemination of findings.

The review suggests that digital health interventions, including web-based programs, instant messaging platforms, web-based personalized normative feedback (PNF), web-based motivational interviewing (MI), and e-health applications, hold promise for reducing substance use among college students. ^59, 60^ However, their effectiveness varies based on factors such as participant motivation, cultural context, and methodological design. Among the targeted population of young adults aged 18 and older, alcohol and tobacco use were the primary focus, reflecting evidence that substance use often peaks between ages 18–25. ^41^ Digital interventions, such as instant messaging and web-based feedback, showed potential in motivating students to abstain from psychoactive substances, though success depended on specific contexts and approaches. Furthermore, substance use disorder represents a major public health challenge with profound impacts on individuals, families, and society. ^61^ Consequently, it is essential to implement substance use reduction programs tailored to this demographic. Due to their cost-effectiveness, accessibility, and ability to reach underserved populations, digital health technologies should be a priority in LMICs to enhance healthcare delivery and expand access to necessary interventions.

While the majority of studies in the review employed quantitative methods, randomized controlled trials (RCTs) were the most common design. Interventions were typically evaluated at both individual and group levels, lending robustness to the findings. However, methodological limitations, such as high attrition rates, reliance on self-reported data, short follow-up periods, and underrepresentation of non-smartphone users, were common. Additionally, few studies explored educational interventions incorporating health literacy and the Health Belief Model, highlighting another research gap. The diverse mechanisms contributing to these significant findings suggest that studies employing eclectic approaches may require replication across different settings to confirm the positive outcomes of digital health interventions in reducing substance use. ^62, 63^

The review identified that digital health interventions for alcohol use were more prevalent than those for other substances, such as tobacco, marijuana, or opioids. This focus is justified by alcohol’s significant contribution to global disease burden and injury outcomes. ^64^ However, studies on interventions for other psychoactive substances should be interpreted cautiously due to the limited data and varying levels of effectiveness. ^65^

Digital health interventions offer significant potential for delivering information and healthcare services aimed at reducing substance use and implementing effective treatment strategies across diverse populations. ^65, 66^ Promising approaches include mobile applications, instant messaging platforms, and web-based programs, which have been shown to reduce stigma, enhance treatment accessibility, and address resource limitations in LMICs. ^67– 69^ Text messaging interventions, in particular, demonstrated affordability and feasibility, making them a practical option for resource-limited settings. ^70, 71^ Despite these advantages, high attrition rates and challenges such as infrastructure limitations, resistance from healthcare staff and associated costs can hinder the acceptability and effectiveness of these interventions. Nevertheless, their overall benefits remain substantial. ^59^

Overall, this review underscores the potential of digital health technologies to reduce substance use among university students in LMICs. Despite challenges such as high attrition rates and methodological constraints, ^65, 72^ these interventions provide innovative, cost-effective, and accessible solutions to address public health concerns. ^73^ Future research should focus on expanding the scope of studies, particularly in underrepresented regions like Africa, and evaluating long-term outcomes to ensure sustainable benefits.

### Strengths and limitations of the review

This scoping review has several notable strengths. It addresses the important topic of digital health interventions for substance use reduction among college and university students in LMICs, utilizing a transparent and reproducible approach for the search strategy, data collection, and extraction processes. The review underscores a significant gap in the literature, revealing that while many studies address substance use, only a small proportion assess the effectiveness of digital health interventions for this specific population.

However, some limitations should be noted. The review relied exclusively English-language journals, potentially omitting relevant studies published in other languages. Furthermore, its aim to provide a broad overview of the existing literature led to the inclusion of studies with diverse methodologies, which may have contributed to the heterogeneity of the reported findings.

## Conclusions

This scoping review offers a thorough overview of the use of digital health technologies to reduce substance use among college and university students in LMICs. It highlights the effectiveness of various digital health interventions and identifies key areas for future research. The findings underscore the significant potential of digital health technologies to address substance use, particularly in underserved communities. When effectively implemented, mobile health interventions could play a pivotal role in reducing substance use. However, despite a growing body of evidence, a notable knowledge gap remains regarding the specific impact of these interventions on LMIC college and university students, making it challenging to definitively establish their effectiveness. To address this gap, larger-scale randomized studies are urgently needed to evaluate these interventions’ efficacy in this population.

Overall, the review highlight the potential of digital, web-based, and mobile interventions to reduce alcohol and smoking behaviors, with their effectiveness varying depending on factors such as participant motivation, cultural context, and intervention design. They emphasize the wide range of strategies, tools, and designs employed in digital interventions for substance use. While many of the interventions in the review showed effectiveness or feasibility, the outcomes differed based on the type of substance, delivery methods, and target populations.

To reduce substance use in low- and middle-income countries (LMICs), a strategic, multi-faceted approach is essential. This involves creating supportive environments for digital health technologies, even in resource-limited settings. Research on substance use reduction should be prioritized, with a focus on disseminating findings widely. Future studies should target college and university students, using larger, more diverse samples, appropriate follow-up periods, and replication across different populations to validate the effectiveness of digital health interventions. Additionally, conducting systematic reviews or meta-analyses will help synthesize existing evidence and improve understanding of mobile health (mHealth) interventions for substance use reduction in these regions. This comprehensive approach will contribute to developing sustainable and effective solutions for the unique challenges in LMICs.

## Ethics and consent

Ethics and consent were not required.

## Data Availability

No data are associated with this article. Figshare repository: Application of digital health technologies to substance use reduction among students in higher education institutions: A scoping review. https://doi.org/10.6084/m9.figshare.28677836.v1 ^74^ This project contains following extended data:
1. PRISMA-ScR-Checklist_2019, S1.pdf 2. Supplementary Materials.pdf 3. Manuscripts.pdf (Table 1 and Table 2) Data are available under the terms of the Creative Commons Zero “No rights reserved” data waiver (CC0 1.0 Public domain dedication).
