# Supplementary material for: Application of digital health technologies to substance use reduction among students in higher education institutions: A scoping review
Source: F1000Res. 2025 Oct 9;14:456. Originally published 2025 Apr 24. [Version 3] doi: 10.12688/f1000research.163565.3 (PMC12589940; doi:10.12688/f1000research.163565.3)
Supplement: Peer review history

# Reviewer report: Reviewer response for version 3

**DOI:** 10.5256/f1000research.189275.r423047

**Published:** 2025-11-05

**Authors:** Glenn Sterner

**Affiliations:** 1 Criminal Justice, University of Kentucky College of Social Work, Lexington, Kentucky, USA

I would like to commend the authors on an important and relevant topic of research for the field, specifically the use of digital technologies to impact substance use behaviors in college aged populations in low and middle income countries.

 In the introduction, first paragraph, the term psychoactive is introduced; this needs to be refined, particularly with regards to the discussion of addiction, dependence and misuse – none of these are particularly indicated by the use of the term psychoactive agnostic of use patterns.  Citing neuroscience literature here would be helpful.

 It is unclear why Ethiopia was called out in paragraph 2 of the introduction.  This should be removed.

 It is unclear if the studies for inclusion in the review were prevention or substance use reduction focused, based on the description in the methodology.  This should be clarified, as there is conflicting discussion in this section of the manuscript.

 It is unclear the time frame of the inclusion of the studies searched – were these studies for all of time?  It becomes critical to understand time frame as the LMIC classification by the world bank was only for 2019-2020; if studies included countries that were in that list in a historical context, they may not be LMICs in previous classifications.  Thus, there must be alignment with the classification of the country as LMIC and the implementation of the intervention within those countries to ensure fidelity of the discussion of effectiveness.

 The third, fourth, and fifth paragraph of the results section are redundant and should be revised.

 Be sure to spell out all acronyms (e.g., AUDIT and ASSESS).

 The section “substance use reduction” does not discuss if the interventions lead to substance use reduction.

 The sections “type of digital health technologies” and “Purpose and digital health strategies” are redundant and should be combined.

 It would be helpful to have a summary table of all of the manuscripts that denotes the intervention, technology, sample size, dose, and outcomes for each paper reviewed.

 The Discussion is repetitive, and it would be strengthened by revising it to reduce redundancies.

Are the rationale for, and objectives of, the Systematic Review clearly stated?

Partly

Is the statistical analysis and its interpretation appropriate?

Yes

If this is a Living Systematic Review, is the ‘living’ method appropriate and is the search schedule clearly defined and justified? (‘Living Systematic Review’ or a variation of this term should be included in the title.)

Not applicable

Are sufficient details of the methods and analysis provided to allow replication by others?

Partly

Are the conclusions drawn adequately supported by the results presented in the review?

Partly

Reviewer Expertise:

Substance use, illicit substance distribution, gambling, human trafficking

I confirm that I have read this submission and believe that I have an appropriate level of expertise to confirm that it is of an acceptable scientific standard, however I have significant reservations, as outlined above.

# Reviewer report: Reviewer response for version 3

**DOI:** 10.5256/f1000research.189275.r421965

**Published:** 2025-10-28

**Authors:** Katarina Ulfsdotter Gunnarsson

**Affiliations:** 1 Linköping University, Linköping, Sweden

I have no further comments.

Are the rationale for, and objectives of, the Systematic Review clearly stated?

Partly

Is the statistical analysis and its interpretation appropriate?

Not applicable

If this is a Living Systematic Review, is the ‘living’ method appropriate and is the search schedule clearly defined and justified? (‘Living Systematic Review’ or a variation of this term should be included in the title.)

No

Are sufficient details of the methods and analysis provided to allow replication by others?

No

Are the conclusions drawn adequately supported by the results presented in the review?

Partly

Reviewer Expertise:

Public health, digital interventions, health eocnomics

I confirm that I have read this submission and believe that I have an appropriate level of expertise to confirm that it is of an acceptable scientific standard.

# Reviewer report: Reviewer response for version 2

**DOI:** 10.5256/f1000research.184204.r401120

**Published:** 2025-08-27

**Authors:** Chunqing Lin

**Affiliations:** 1 University of California, Los Angeles, CA, USA

This article reviews current digital interventions to prevent and reduce substance use among college students in low- and middle-income countries. The topic is timely and important. I have the following comments to improve clarity and rigor:

1. Please explain the rationale for including non-RCT designs (e.g., qualitative studies, cross-sectional designs) given the stated objective of exploring effectiveness. Note that intervention effectiveness cannot be evaluated in a cross-sectional study.
2. The definition of digital intervention is not clear. Was a predefined definition of "digital intervention" established prior to the review? The Methods section lists examples (“such as mobile apps, web-based interventions, and SMS”), but it is unclear whether other formats, such as telehealth services, wearable devices, or other emerging technologies, were excluded by design or simply did not emerge from the scoping review.
3. Please clarify whether article screening and data extraction were conducted by at least two independent reviewers, and whether double data extraction was performed.
4. For the included studies, note that Hong Kong and South Korea are not classified as LMICs under World Bank criteria. Please clarify why they were included and whether this aligns with the stated inclusion criteria.
5. In terms of study populations, in the paragraph describing sample sizes, it would be informative to clarify whether participants were general college students, those at higher risk, or individuals already engaging in substance use. In other words, please define the population characteristics more explicitly.
6. There is some repetition in the Results section that could be streamlined to improve readability.
7. A single table summarizing key characteristics of the included studies, such as study design, country, study population (with sample size), type of digital health intervention, targeted substances, and main outcomes, would greatly enhance clarity and allow readers to compare studies more easily.

Are the rationale for, and objectives of, the Systematic Review clearly stated?

Yes

Is the statistical analysis and its interpretation appropriate?

Not applicable

If this is a Living Systematic Review, is the ‘living’ method appropriate and is the search schedule clearly defined and justified? (‘Living Systematic Review’ or a variation of this term should be included in the title.)

Not applicable

Are sufficient details of the methods and analysis provided to allow replication by others?

Partly

Are the conclusions drawn adequately supported by the results presented in the review?

Yes

Reviewer Expertise:

Substance use, digital health, HIV, behavioral intervention

I confirm that I have read this submission and believe that I have an appropriate level of expertise to confirm that it is of an acceptable scientific standard, however I have significant reservations, as outlined above.

# Response

**Published:** 2025-10-01

**Authors:** Mistire Teshome Guta

**Affiliations:** Jimma University, Jimma, Oromia, Ethiopia

Point by point response

 First we like to thank the reviewer for his supporting comments and time.

 This article reviews current digital interventions to prevent and reduce substance use among college students in low- and middle-income countries. The topic is timely and important. I have the following comments to improve clarity and rigor:

1. Please explain the rationale for including non-RCT designs (e.g., qualitative studies, cross-sectional designs) given the stated objective of exploring effectiveness. Note that intervention effectiveness cannot be evaluated in a cross-sectional study.

R_ First of all we like to thank our respected reviewer for his/her important observation. We are thinking that the term ‘effectiveness’ is too narrow and didn’t align with our inclusion for the scoping review.  So, we primarily aim in the scoping review to map the existing literatures in order to identify the key concepts, evidence gaps, types of available research. So, critically appraising and synthesizing the evidences for effectiveness is the goal of systematic review and meta-analysis.  The effectiveness that we use in the methods section was as one of the outcomes that we charted and it wasn’t used as a criterion for the inclusion.

 We believe that the qualitative studies and some cross-sectional studies are very important to provide insight to acceptability, feasibility and user perception for the digital intervention. So, we revise our objective:  mapping and summarizing existing literature on types of digital health technologies used, their intended purposes, and reported outcomes that targeting substance use reduction among college students in low- and middle-income countries (LMICs).

1. The definition of digital intervention is not clear. Was a predefined definition of "digital intervention" established prior to the review? The Methods section lists examples (“such as mobile apps, web-based interventions, and SMS”), but it is unclear whether other formats, such as telehealth services, wearable devices, or other emerging technologies, were excluded by design or simply did not emerge from the scoping review.

R_ Yes, we adapt the definition from the WHO definition of the digital health intervention: ‘the digital use, mobile and wireless technology for the achievements of the health objectives.’ In our inclusion criteria we list technologies like mobile apps, web-based interventions, and SMS but we didn’t excluded emerging technologies like wearable devices. Due to the current state on the published literature on our specific topic, we easily get more accessible technologies like SMS and web portals.

1. Please clarify whether article screening and data extraction were conducted by at least two independent reviewers, and whether double data extraction was performed.

R_ We would like to apologize for not reporting in the manuscript. In the screening process the titles, abstract and full texts were screened by two independent reviewers (MTG and FA) and any disagreements resolved through discussion among the reviewers or by consulting the third reviewer (KHA). Data extraction was done by two independent reviewers (MTG and DA) through using standardized data extraction form. The third reviewer (FA or KHA) were involved accordingly.

1. For the included studies, note that Hong Kong and South Korea are not classified as LMICs under World Bank criteria. Please clarify why they were included and whether this aligns with the stated inclusion criteria.

R_  Sorry for our mistake and We remove the two studies from Hong Kong (Chau et al., 2023; Chau et al., 2024) and the one South Korea (Sharifi et al., 2019) then we run our analysis with 8 included studies.

1. In terms of study populations, in the paragraph describing sample sizes, it would be informative to clarify whether participants were general college students, those at higher risk, or individuals already engaging in substance use. In other words, please define the population characteristics more explicitly.

R_ We will revise the ‘Characteristics of the studies’ subsection to explicitly grouped the participants for each study through clarifying about the general student population , at risk students and those participants engaged in the substance use.

1. There is some repetition in the Results section that could be streamlined to improve readability.

R_ Accepted and done.

1. A single table summarizing key characteristics of the included studies, such as study design, country, study population (with sample size), type of digital health intervention, targeted substances, and main outcomes, would greatly enhance clarity and allow readers to compare studies more easily.

R_ Accepted and done.

# Reviewer report: Reviewer response for version 1

**DOI:** 10.5256/f1000research.179938.r391262

**Published:** 2025-06-20

**Authors:** Katarina Ulfsdotter Gunnarsson

**Affiliations:** 1 Linköping University, Linköping, Sweden

This scoping review investigates the use of digital health technologies to reduce substance use among college and university students, particularly in low- and middle-income countries (LMICs). The review analyzed 11 studies covering interventions in seven countries.

 First, I would like to see a clear definition of substance use already in the abstract stating what you include in the term. For example do this involves alcohol? Tobacco? Please also further develop this in the introduction.

 Additionally, clarify in the abstract that the focus of the scoping review is on LMICs. Currently, it may be interpreted as a finding rather than the study's focus.

 Make sure the purpose of the study aligns with the method and the rest of the article. Review this section.

 Could you please state the inclusion and exclusion criteria in the Methods section? For instance, how did you handle studies that focused on interventions targeting more than one substance, such as physical activity, diet, alcohol, and smoking? The inclusion and exclusion criteria should be clearly outlined. Were there any specific criteria for who would deliver the intervention? What outcomes did you consider? Did you only include randomised controlled trials? Were there any restrictions on the control group? Were there any restrictions on the publication year?

 According to the research question, the review aims to explore *the effectiveness* of reducing substance use. Therefore, it's essential to clearly explain why you've included various study designs, such as a qualitative study, in the method section.

 The fourth paragraph in the Characteristics of the studies repeats the first. Please remove it this.

 Please consider a table to summaries the characteristics of studies/outcomes dose and duration / purpose / substance use reduction and type of digital health technologies. The results are presented in several sections, which renders it somewhat challenging to follow each individual study. It would be more advantageous to present the studies sequentially. Additionally, comprehending the outcomes measured is difficult without knowledge of the study design and the objectives of each study. I would recommend creating a table to effectively present the 11 studies.

 In the discussion, you state, “Of the few studies identified, only four focused on LMICs..”. This is also an indication that the inclusion criteria need to be clarified. Otherwise, it is difficult to understand the purpose of the review. If you didn't include only studies from LMICs, I find it hard to believe you found just 11.

 The limitation is rather short and I would like to see further discussions on the limitations of the review.

Are the rationale for, and objectives of, the Systematic Review clearly stated?

Partly

Is the statistical analysis and its interpretation appropriate?

Not applicable

If this is a Living Systematic Review, is the ‘living’ method appropriate and is the search schedule clearly defined and justified? (‘Living Systematic Review’ or a variation of this term should be included in the title.)

No

Are sufficient details of the methods and analysis provided to allow replication by others?

No

Are the conclusions drawn adequately supported by the results presented in the review?

Partly

Reviewer Expertise:

Alcohol, randomised controlled trial, health economic evaluations, systimatic reviwes

I confirm that I have read this submission and believe that I have an appropriate level of expertise to state that I do not consider it to be of an acceptable scientific standard, for reasons outlined above.

# Response

**Published:** 2025-06-26

**Authors:** Mistire Teshome Guta

**Affiliations:** Jimma University, Jimma, Oromia, Ethiopia

Point-by-point response to the reviewer (Katarina Ulfsdotter Gunnarsson, **Linköping University, Linköping, Sweden)**

 Thank you for taking the time to review our scoping review and for providing such support and valuable feedback. Below, we have addressed your comments and questions in a point-by-point response.

 1. First, I would like to see a clear definition of substance use already in the abstract stating what you include in the term. For example, does this involve alcohol? Tobacco? Please also further develop this in the introduction.

**R_** Accepted, and the abstract is updated. Yes our review includes alcohol, marijuana, nicotine, and khat. We also include the definition in the *Introduction *part, paragraph *1:*

***‘*** *Psychoactive substances, which include compounds that alter mental processes such as perception, consciousness, cognition, mood, and emotions, encompass a wide range of substances like alcohol, marijuana, nicotine, and khat.* ***’***

 2. Additionally, clarify in the abstract that the focus of the scoping review is on LMICs. Currently, it may be interpreted as a finding rather than the study's focus.

**R_**  Accepted and the abstract is updated accordingly.

***‘*** *Psychoactive substances alter perception, mood, cognition, or consciousness and include a wide range of compounds such as alcohol, marijuana, nicotine, and khat. Substance use among college and university students is associated with significant health issues, academic struggles, and premature death. This scoping review examines digital health interventions, including mobile and internet platforms, targeting substance use reduction among college students in low- and middle-income countries (LMICs). A comprehensive search across databases such as PubMed, PsycINFO, Scopus, and Google Scholar identified 11 eligible studies conducted across seven countries between 2013 and 2025. These studies focused primarily on alcohol use and included digital health tools like instant messaging, Telegram applications, text messaging, and web-based interventions. The results suggest that digital health technologies can effectively motivate college students in LMICs to reduce or abstain from psychoactive substance use. However, there is a notable research gap in evaluating the effectiveness and feasibility of these tools, especially mobile text messaging, which remains one of the most widely used methods in LMICs. The review highlights the need for further research, including systematic reviews and meta-analyses, to better understand the impact of digital health interventions on substance use reduction and to develop evidence-based programs for behavior change.* ***’***

 3. Could you please state the inclusion and exclusion criteria in the Methods section? For instance, how did you handle studies that focused on interventions targeting more than one substance, such as physical activity, diet, alcohol, and smoking? The inclusion and exclusion criteria should be clearly outlined. Were there any specific criteria for who would deliver the intervention? What outcomes did you consider? Did you only include randomised controlled trials? Were there any restrictions on the control group? Were there any restrictions on the publication year?

 R_  the inclusion and exclusion criteria, it is accepted and updated in *methods *part:

*‘* *The inclusion criteria for this review focused on studies involving higher education students (university or college) from low- and middle-income countries (LMICs), as classified by the World Bank for 2019–2020. Eligible studies evaluated digital health technologies—such as mobile apps, web-based interventions, and SMS—designed to reduce substance use, including alcohol, tobacco, marijuana, and khat. Studies needed to report outcomes related to substance use prevention, harm reduction, or cessation within LMIC contexts. All study designs were considered, including quantitative, qualitative, mixed methods, randomized controlled trials (RCTs), quasi-experimental, and observational studies. Only original studies published up to March 2025 were included, with no restriction on the earliest year of publication.*

*Exclusion criteria ruled out studies conducted in high-income countries or those not focused on LMIC students. Studies using solely non-digital interventions, such as face-to-face counseling or paper-based methods, were excluded. Also excluded were studies that did not report substance use-related outcomes—for instance, those focused only on physical activity or diet. Non-peer-reviewed literature, including protocols, editorials, commentaries, conference abstracts (unless full data were provided), and systematic reviews, were not considered, although their references were screened for potential inclusion. Multi-component interventions were included only if the digital health component’s effect could be isolated or was clearly the primary focus of the study.* *’*

- *Interventions Targeting Multiple Behaviors: Studies were included if they measured substance use outcomes, even if they also addressed other behaviors (e.g., physical activity + alcohol use).  *
- *Intervention Deliverers: No restrictions were placed on who delivered the intervention (e.g., researchers, healthcare providers, automated systems).  *
- *Control Groups: No restrictions were applied (e.g., no intervention, active control, or waitlist).  *
- *Outcomes Considered: Any substance use-related outcome (e.g., reduction in frequency/quantity, abstinence, behavioral change).  *
- *Rationale for Inclusivity: Since this was a scoping review, the goal was to map all available evidence, so no studies were excluded based on quality (though quality was assessed using JBI tools).  *

4. According to the research question, the review aims to explore *the effectiveness* of reducing substance use. Therefore, it's essential to clearly explain why you've included various study designs, such as a qualitative study, in the method section.

 R_  The research question was developed to explore the effectiveness of digital health technologies for substance use reduction among higher education students in LMICs, examining the technologies used, their key features, and their outcomes. Recognizing that "effectiveness" encompasses both quantitative outcomes and implementation factors, we intentionally included diverse study designs (RCTs, quasi-experimental, qualitative, and mixed-methods studies) in our review. This approach allowed us to: (1) capture quantitative evidence of behavior change while also understanding contextual factors through qualitative insights; (2) address evidence gaps in LMICs where rigorous experimental studies may be limited; and (3) comply with scoping review methodology that emphasizes comprehensive evidence mapping over restrictive design criteria. By incorporating multiple study types, we gained a more complete understanding of how these interventions work in real-world settings, including user engagement, cultural adaptation, and barriers to implementation - all critical components for assessing true effectiveness in the target population.

 5. The fourth paragraph in the Characteristics of the studies repeats the first. Please remove it this.

 R_ accepted and paragraph one is updated according to your comment.

*‘* *The review identified 11 original, peer-reviewed studies (published between 2015 and 2025) examining digital health interventions for substance use reduction in LMICs. Publication trends revealed a gradual increase in research interest, with five studies (45.5%) published in the last five years (2020–2025). The year 2019 had the highest number of publications (n = 3, 27.3%), followed by 2018, 2023, and 2024 (two studies each, 18% per year), while 2015 and 2020 contributed one study each. Despite this growth, the limited total number of studies highlights a significant research gap in LMICs. The earliest eligible study dated back to 2015* *( 43)* *, the earliest eligible study dated back to 2015* *( 44, 45)* *, 2019* *( 41, 46, 47)* *, 2020* *( 48)* *, 2023* *( 42, 49)* *and 2024* *( 50, 51)* *(Figure 2).* *’*

 6. Please consider a table to summaries the characteristics of studies/outcomes dose and duration / purpose / substance use reduction and type of digital health technologies. The results are presented in several sections, which renders it somewhat challenging to follow each individual study. It would be more advantageous to present the studies sequentially. Additionally, comprehending the outcomes measured is difficult without knowledge of the study design and the objectives of each study. I would recommend creating a table to effectively present the 11 studies.

 R_ Would you mind checking Table 1 at https://doi.org/10.6084/m9.figshare.28677836 ? It summarizes all of your concerns clearly.

 7. In the discussion, you state, “Of the few studies identified, only four focused on LMICs..”. This is also an indication that the inclusion criteria need to be clarified. Otherwise, it is difficult to understand the purpose of the review. If you didn't include only studies from LMICs, I find it hard to believe you found just 11.

 R_Please consider it as a type error. We correct it like:

*‘The findings reveal a notable lack of peer-reviewed articles on this topic, especially from 2020 to 2025, with only five studies identified and none from Africa, underscoring a critical research gap.* *’*

 8. The limitation is rather short and I would like to see further discussions on the limitations of the review.R_ accepted and updated accordingly.

 ‘ ***Strengths and Limitations***

*This scoping review provides a valuable synthesis of digital health interventions for substance use reduction among university students in LMICs, employing a rigorous methodology guided by PRISMA-ScR standards. Its key strengths include a comprehensive search strategy across multiple databases, clearly defined inclusion criteria, and a focus on an under-researched population in global health. However, several important limitations must be acknowledged. The review was restricted to English-language publications, potentially excluding relevant studies in other languages and introducing geographic bias, as evidenced by the predominance of studies from Brazil and absence of research from Africa. The included studies exhibited significant heterogeneity in design, interventions, and outcome measures, which, while providing breadth of understanding, limited direct comparisons of effectiveness. Most concerning is the paucity of studies (only 11 over a decade) meeting inclusion criteria, suggesting either limited primary research in LMICs or publication bias favoring high-income country research.*

*Methodological challenges were apparent across studies, including short follow-up periods (typically ≤6 months), high attrition rates, and reliance on self-reported data, all of which constrain conclusions about long-term effectiveness. The predominance of alcohol-focused interventions (7 of 11 studies) leaves other substance use behaviors underexplored. Technological barriers were rarely addressed, despite varying internet access across LMICs potentially limiting intervention reach. While maintaining broad inclusion criteria aligned with scoping review methodology, the incorporation of studies with varying quality levels may affect overall findings' robustness.*

*These limitations highlight critical gaps for future research, including the need for culturally adapted interventions in underrepresented regions, longer-term evaluations incorporating objective measures, and studies examining implementation barriers in low-resource settings. Addressing these challenges will be essential for developing effective, scalable digital solutions to reduce substance use in LMIC university populations.  *’
